# Developing resources to support the diagnosis and management of Chronic Fatigue Syndrome/Myalgic Encephalitis (CFS/ME) in primary care: a qualitative study

**DOI:** 10.1186/1471-2296-13-93

**Published:** 2012-09-21

**Authors:** Kerin Hannon, Sarah Peters, Louise Fisher, Lisa Riste, Alison Wearden, Karina Lovell, Pam Turner, Yvonne Leech, Carolyn Chew-Graham

**Affiliations:** 1 School of Community Based Medicine, University of Manchester, Manchester, UK; 2 School of Psychological Sciences, University of Manchester, Manchester, UK; 3 National School for Primary Care Research, University of Manchester, Manchester, UK; 4 School of Nursing, Midwifery and Social Work, University of Manchester, Manchester, UK; 5 School of Community Based Medicine and National School for Primary Care Research, University of Manchester, Manchester, UK

**Keywords:** Chronic fatigue syndrome, ME, Resources, Patient, Practitioner, Qualitative research, Primary health care

## Abstract

**Background:**

NICE guidelines emphasise the need for a confident, early diagnosis of Chronic Fatigue Syndrome/ Myalgic Encephalitis (CFS/ME) in Primary Care with management tailored to the needs of the patient. Research suggests that GPs are reluctant to make the diagnosis and resources for management are currently inadequate. This study aimed to develop resources for practitioners and patients to support the diagnosis and management of CFS/ME in primary care.

**Methods:**

Semi structured interviews were conducted with patients, carers, GPs, practice nurses and CFS/ME specialists in North West England. All interviews were audio recorded, transcribed and analysed qualitatively using open explorative thematic coding. Two patient involvement groups were consulted at each stage of the development of resources to ensure that the resources reflect everyday issues faced by people living with CFS/ME.

**Results:**

Patients and carers stressed the importance of recognising CFS/ME as a legitimate condition, and the need to be believed by health care professionals. GPs and practice nurses stated that they do not always have the knowledge or skills to diagnose and manage the condition. They expressed a preference for an online training package. For patients, information on getting the most out of a consultation and the role of carers was thought to be important. Patients did not want to be overloaded with information at diagnosis, and suggested information should be given in steps. A DVD was suggested, to enable information sharing with carers and family, and also for those whose symptoms act as a barrier to reading.

**Conclusion:**

Rather than use a top-down approach to the development of training for health care practitioners and information for patients and carers, we have used data from key stakeholders to develop a patient DVD, patient leaflets to guide symptom management and a modular e-learning resource which should equip GPs to diagnose and manage CFS/ME effectively, meet NICE guidelines and give patients acceptable, evidence-based information.

## Background

Chronic Fatigue Syndrome (CFS) or Myalgic Encephalitis (ME) is characterized by severe, disabling, medically unexplained fatigue that is not alleviated by rest and lasts at least six months [1,2]. Patients complain of concentration or memory problems, disturbed sleep, weakness and pain, and symptoms differ between patients [2]. CFS/ME is associated with a high level of distress and disruption to patients lives, and is costly in terms of both health service utilization and economic burden to patients and their families [3-5].

Diagnosis of CFS/ME is formulated through the patient history and exclusion of other medical causes and psychiatric problems [2]. Patients have described how receiving a diagnosis of CFS/ME is the single most helpful event for them in managing their condition [6]. NICE guidelines also emphasise the importance of an early diagnosis [1]. However, in reality this rarely happens, and some GPs remain sceptical about the diagnosis [7-10]. In a recent primary care treatment trial [11], the mean time from onset of symptoms to diagnosis was 3.6 years. It is likely therefore that at any one time a number of individuals are awaiting diagnosis, suggesting the UK prevalence of CFS/ME of 0.2-0.4% [12] is likely to be a low estimate.

Despite NICE recommendations for early treatment and developing tailored care-packages [1], this rarely happens in practice [13]. CFS/ME is not incentivised as part of the Quality and Outcomes Framework (QOF), a pay-for-performance scheme that financially rewards GP practices for achieving a number of clinical and organisational indicators [14]. The complexity of the condition and lack of incentives can therefore mean that the diagnosis and management of symptoms of diseases not included in the QOF can be overlooked [15,16]. For example, 65% of the Action for ME members surveyed [17] report never receiving any treatment. Even when a diagnosis has been given, many primary care health professionals feel ill equipped to deliver treatments [18,19]. The need for better communication and education on the management of CFS/ME has been consistently highlighted [18,20,21]. In order to meet NICE guidelines, more usable and accessible educational resources are needed for patients and their primary care team [6]. There is also a need to develop culturally sensitive information as Bhui et al. [22] report that ethnic minority groups may have a higher prevalence of CFS/ME than White groups, but remain undiagnosed and untreated.

This paper presents the initial phase of the METRIC (ME Education, Training and Resources In Primary Care) study that links with the Medical Research Council framework for complex interventions [23]. The study aims to develop an education and training intervention to support practitioners in making an early diagnosis of CFS/ME and supporting patients in the management of their symptoms. Patient and carer perspectives were integral to the development of the resources, with input directly into the research management group, patient involvement groups and qualitative interviews.

## Methods

### Ethics

Ethical approval was obtained from the Greater Manchester East Ethics Committee (10/H1001/5). R&D approval was granted by NHS Manchester, Central Lancashire, Stockport and Bury.

### Patient and public involvement

The patient co-investigator and carer attended bi-monthly research management team meetings and provided input and feedback on the resources at each stage of development. Two patient involvement groups (consisting of patients and carers) met three times to provide feedback on the development, content, presentation and acceptability of the resources.

### Design

Semi-structured interviews were conducted with patients and carers, GPs and practice nurses and Specialist staff from CFS/ME services.

### Sampling

#### Patients and carers

Patients and carers were recruited using a number of methods: advertisement, through existing CFS/ME support groups, community groups and via the patient co-investigator (PT). Other participants were recruited through specialist CFS/ME services in the NHS in response to a project flyer. In order to ensure the resources developed were culturally sensitive, a purposive sample of BME (black and minority ethnic) patients were also recruited from South Asian third sector groups in Greater Manchester using flyers and personal visits to community groups.

#### Practitioners

A purposive sample of GP Practices were recruited by personal invitation and at two GP education meetings. A flyer outlined the aims of the study and gave the research team’s contact details for those who wanted further information. We attempted to sample on size of practice, demographics (inner city/urban/sub-urban; ethnic mix) and training practice. Practice nurses were recruited by a variety of methods which our previous experience suggests is needed [24]. Firstly all GPs interviewed were asked to forward details of the study on to practice nurses in their practice. Secondly, a project flyer was sent out to all lead practice nurses in two Primary Care Trusts (PCTs), inviting them to participate and asking them to forward the details on to the rest of their practice nurse forum. Finally, one practice nurse was recruited through a contact with one patient involvement group.

#### Specialist CFS/ME practitioners

A letter of invitation and an information sheet were sent to four CFS/ME specialists working in local CFS/ME services inviting them to participate in a semi-structured interview.

### Data collection

Individual semi structured interviews were conducted face-to-face with 9 GPs, 5 Practice Nurses, 4 CFS/ME specialists, 10 carers and 16 patients (including 12 BME patients and carers) during 2011–2012 (Tables 1 and 2).Topic guides (available from the authors) were developed from a review of the literature and research team discussions. Patient/carer interviews focussed on experiences of being diagnosed, support received in primary care, carers’ needs, and resource needs to enable them to manage their condition. Modes of delivery of information were discussed with reference (verbally) to existing patient information materials. Practitioner interviews focussed on current practice in the diagnosis and management of patients with CFS/ME, attitudes towards CFS/ME and training and education needs in order to be confident in making the diagnosis of CFS/ME and manage patients effectively. Specialist CFS/ME practitioner interviews focussed on the needs of patients and asked for comments on existing CFS/ME resources. In addition, practitioner respondents were invited to discuss resources and educational needs in primary care professionals and suggestions about mode of delivery, with reference to their usual ways of learning.

**Table 1:** Patient and carer demographics

**ID number**	**Gender**	**Age**	**Ethnicity**	**Severity of CFS/ME (self-defined)**	**Number of years since diagnosis**
**Patient 1**	Female	42	White British	Severe	9
**Patient 2**	Male	50	White British	Moderate/severe (now mild)	3
**Patient 3**	Male	61	Indian	Severe	22
**Patient 4**	Female	28	White British	Mild	1.5
**Patient 5**	Female	39	White British	Mild/moderate	0.5
**Patient 6**	Female	37	South Asian	Moderate/severe	7
**Patient 7**	Male	46	White British	Mild/moderate	7
**Patient 8**	Female	64	White British	Severe	20
**Patient 9**	Female	52	South Asian	Mild/moderate	Self-diagnosed
**Patient 10**	Female	32	Other white	Mild/moderate	2
**Patient 11**	Male	35	Indian	Moderate (recently recovered)	1
**Patient 12**	Female	38	South Asian	Mild/moderate	2.5
**Patient 13**	Female	48	South Asian	Moderate	2
**Patient 14**	Male	38	South Asian	Mild	6
**Patient 15**	Female	40	Black British	Moderate	3
**Patient 16**	Female	39	Black British	Mild	Self-diagnosed
**Carer 1**	Female	66	White British	Cares for daughter: severe	16
**Carer 2**	Female	53	White British	Cares for son: moderate	4
**Carer 3**	Female	45	White British	Cares for husband: severe	6
**Carer 4**	Female	47	White British	Cares for daughter: mild/moderate	5
**Carer 5**	Female	57	Indian	Cares for husband: severe	22
**Carer 6**	Male	58	White British	Cares for wife: moderate/severe	10
**Carer 7**	Male	70	White British	Cares for wife: moderate/severe	22
**Carer 8**	Male	58	White British	Cares for wife: moderate	12.5
**Carer 9**	Male	46	White British	Cares for wife: moderate	2
**Carer 10**	Male	71	South Asian	Cares for his wife: moderate/severe	8

**Table 2:** Health care professional details

**Health Professional ID**	**Gender**	**Number of years qualified**	**List size**
**GP 1**	Female	24	Medium
**GP 2**	Male	24	Medium
**GP 3**	Female	14	Medium
**GP 4**	Female	10	Large
**GP 5**	Female	6	Small
**GP 6**	Male	8	Medium
**GP7**	Female	8	Large
**GP8**	Female	16	Medium
**GP9**	Male	8	Medium
**Practice nurse (PN) 1**	Female	8	Medium
**Practice nurse (PN) 2**	Female	8	Medium
**Practice nurse (PN) 3**	Female	5	Small
**Practice nurse (PN) 4**	Female	20	Large
**Practice nurse (PN) 5**	Female	12	Medium
**CFS/ME Specialist 1 (physiotherapst)**	Female	20 (worked in CFS/ME 10 years)	n/a
**CFS/ME Specialist 2 (specialist nurse)**	Female	22 (worked in CFS/ME field 13 years)	n/a
**CFS/ME Specialist 3 (specialist nurse)**	Female	18 (worked in CFS/ME 10 years)	n/a
**CFS/ME Specialist 4 (physiotherapst)**	Male	20 (worked in CFS/ME 8 years)	n/a

### Analysis

Interviews were digitally recorded and transcribed verbatim. Analysis was conducted in parallel with the interviews and was initially inductive, using thematic analysis [25] that were in line with modified grounded theory [26]. Thematic categories were identified in initial interviews and then explored in subsequent interviews. Disconfirming evidence was actively sought and was used to modify emerging themes. Main categories were then compared across interviews and reintegrated into common themes [26,27]. Interview transcripts were read, annotated, and categorised independently by researchers of different professional backgrounds (Psychology, Primary Care, Nursing) to increase reliability of the analysis [28]. Open coding was used rather than axial or selective coding [29] initially. The authors agreed theoretical saturation across the data sets was achieved when no new themes emerged during the final interviews. Because of the limited number of CFS/ME practitioners employed in the PCTs under study, category saturation was not achieved in this data set, but we believe this data set is valuable in adding to the total data, giving a further perspective on resource development.

When the data set had been fully analysed, a deductive approach was taken, with an emphasis on themes relevant for resource development.

## Results

The results presented here focus on the key issues for the development of practitioner resources and training around the diagnosis and management of CFS/ME. Five themes were identified: 1. the need to ensure that the patient feels believed; 2. the importance of a positively framed diagnosis; 3. defining, prioritising and managing symptoms; 4. getting the most out of a consultation and 5. the role of carers. Illustrative quotes are provided and the unique identifier is provided in parentheses. For each theme, the implications for resource development were also examined in the corresponding data.

### The need to be believed

Patients and carers described their frustration when GPs or practice nurses did not recognise the seriousness of their symptoms or questioned the legitimacy of the condition:

*“he was really, really horrible, and he just said…he doesn't not acknowledge that people get ME, but he sat there and he said, 'I think we all get ME at some point in our lives. . So I changed doctors because I thought how can you be treated (like that)?”* Patient 1 (female, severe CFS/ME).

Patients reported feeling that there had been an element of luck in achieving a diagnosis of CFS/ME and reported receiving recommendations from other patients of sympathetic health professionals who saw their symptoms as representing a legitimate illness:

*“The manager (at the acupuncture clinic)… she was a very lovely lady, and she goes, do one thing, go to Doctor [names GP]… So we went to that surgery. It is quite far off… (But) even now there are GPs in that same surgery who says so you think that Doctor [names GP] is going to get you better? They don’t believe in it.”* Patient 3 (male, severe CFS/ME).

When each test came back as negative, patients described feeling that their condition was invisible, and they were at fault for ‘imagining’ or ‘causing’ their symptoms:

“*GPs are the gatekeepers to the specialist services, and if they’re actually saying there’s nothing wrong with her that a good night’s sleep won’t cure, then what can you do. So she started to feel that maybe it was in her head and she felt really depressed about it all… he says he doesn’t know so there’s no point in going back.”* Carer 1 (female, daughter with severe CFS/ME).

Patients who felt believed by their GP described how important this was to their wellbeing. A diagnosis allowed them to tell family, friends and their employer that they had a label for their symptoms:

“*when it was diagnosed formally, that was actually a big relief for me, because at least I knew that finally I could tell people what I had. Because previously, when I talked to people, I’d tell them I was ill and they said, “What have you got?” I said, “I don’t know”. And so this was a big…I think, it was a big pressure off my mind, because I’d been having all these symptoms, I didn’t know what it was, nobody understood what they were.. eventually when she formally diagnosed (me), that was a big, big step forward.”* Patient 14 (male, mild CFS/ME).

The GP and practice nurse respondents expressed varying degrees of understanding of CFS/ME and some questioned whether CFS/ME was a legitimate illness. These health professionals were unaware of the evidence base for this condition or believed that the symptoms of CFS/ME could be explained by a psychological problem or secondary gain:

*“I don’t use the term ME because I think it’s completely without evidence… I’d want to check that they’re not depressed or anxious or in a state of secondary gain.”* GP 1.

*“there’s still a bit of controversy around as to whether it actually exists… I guess there’s a part of me that thinks is it something that could be used by patients that want an excuse not to work… as the nurse you’d feel there’s nothing really I can do”* PN1.

Those GPs and practice nurses who did recognise CFS/ME as a legitimate illness were aware that some of their colleagues fail to identify this condition which can lead to an inappropriate diagnosis:

“the problem I think lies in the fact that not all GPs are willing to accept or are aware of its existence, and they don't like people dropping in with tiredness all the time, time and time again. And so they get put on antidepressants, sleeping pills, blah-blah-blah, and you can manage your own thing.” GP 6.

#### Implications for resource development

Across patient and health professional interviews there was acknowledgement of the value of GPs recognising CFS/ME as a legitimate condition and to have a sound understanding of the condition. This highlights a need for training to challenge the unsympathetic attitudes held by some practitioners. However, some GPs and practice nurses were hesitant to prioritise time for face-to-face training due to the low priority given to CFS/ME:

*“I don’t think it’s seen as a major illness that’s worth money and QOF points…people will do stuff if they think there’s a financial incentive in it… So no, I don’t think they would prioritise it and I don’t think it would be something that they’d (other GPs in practice) want to have a meeting about.”* GP 7.

However, health professionals suggested that they might engage in training if they thought that it would help them to reduce the number of consultations with patients who repeatedly present with symptoms of CFS/ME. Practitioners described the need for easily accessible IT based training. An e-learning module was therefore believed to be the optimal approach.

### Framing the diagnosis positively

All patients highlighted the need for the diagnosis to be given by the GP in a positive way to maintain hope that symptoms can improve:

“*That was like… it’s like the sun coming out basically. It was so wonderful that she could see a consultant and physio and counsellor, everybody really that she needed to see.”* Carer 1 (female, daughter with severe CFS/ME).

Patients described having been given a diagnosis of CFS/ME without any advice on symptom management or support:

*“it wasn’t positive, he said basically there’s nothing else. I’ll diagnose it as chronic fatigue, but I don’t really want to label you as a person with ME. I said, well, for me, I need some diagnosis for work, I said, because I cannot carry on being off and he said, well my diagnosis will be the chronic fatigue if you want it… I felt like I’ve been given a life sentence. There was no, “Well you could get better” or anything like that, it was terrifying… (Patients need) more about the condition, that people can recover from it rather than finding it depressing that that is it, you’ve got it, and you’re always going to have it. I’m sick of getting told that.”* Patient 5 (female, mild/moderate CFS/ME).

Practitioners described how the diagnosis of CFS/ME was made by exclusion, due to the lack of positive diagnostic criteria. Some GPs and practice nurses used the label as a last resort and with some reluctance due to their own lack of knowledge, but also because making the diagnosis did not lead to obvious treatment approaches:

“*I think I would struggle to diagnose…* [it would be better] *if I had more awareness of what the diagnostic criteria were for chronic fatigue syndrome but in my experience it’s what people tend to get labelled with if anything else can’t be found… I certainly wouldn’t feel confident, I wouldn’t feel that I’d know enough to treat chronic fatigue syndrome myself”* PN1.

Some GPs and practice nurses stated that they avoided the diagnosis as they believed that there was no cure for CFS/ME or that the label may be harmful and act to exacerbate the symptoms:

*“I don’t really feel I know enough about it to tell them an awful lot because I’m not convinced it’s that positive, the things that we can do for them really is it? I’m not convinced it’s a diagnosis that you can be making that is massively going to help somebody.”* GP 7.

However, CFS/ME specialists and other GPs did recognise the importance of a positively framed diagnosis, and the impact this can have on the patient’s quality of life:

*“Actually making a diagnosis can be quite empowering for patients as long as all of the causes have been excluded, that all red flags have been excluded, et cetera, and that the clinical history sort of makes sense, the story, listening to the patient’s story, when you’ve heard it many times it seems fairly obvious but you actually need to make time to hear it.”* CFS/ME specialist 2.

#### Implications for resource development

It was evident from across the groups that a positively framed diagnosis is valued and has implications for management. The practitioner needs to discuss the possibility of CFS/ME in a positive manner, providing patients both with an explanation for their fatigue and with options for symptom management. The use of a template built into the GP computer system was suggested as being potentially useful to aid practitioners to conduct a symptom check-list and carry out the necessary investigations and view management options.

The data highlight the need for a tool for use in primary care to aid a positive diagnosis. CFS/ME specialists currently use an activity diary to record a patient’s level of fatigue and the impact of everyday tasks on symptoms. This diary could be used to facilitate coming to a positive diagnosis and highlight the severity of the patient’s symptoms allowing the patient to demonstrate to the GP how fatigue varies, particularly how fatigue can be prolonged and recovery slow.

This suggests that the e-learning resource for GPs needs to include a computer template to assist the diagnostic process, guidance on how to use an activity diary to aid a positive diagnosis and perhaps a video-recording of a consultation in which the diagnosis is being framed to the patient in a positive manner.

### Defining, prioritising and managing symptoms

CFS/ME is a complex condition and patients described a variety of symptoms and co-morbidities. Patients commented on how it can be difficult to describe the extent and impact of their symptoms to their GP. They described visiting their GP on ‘a good day’, when they had the energy to leave the house:

*“I think one thing between me and my GP is complete lack of awareness of how I am when I'm really ill… if I can't see him I'll cancel appointments, cancel appointments until I'm fit enough to go in, to walk in and sit there and see him. So he only ever sees me when I'm capable of being like that. So I sit there like this and I'll go, 'I've got ME.' But it's not my typical bad day or day of ME, and (when) it's like this it will exhaust me talking to you”* Patient 1 (female, severe CFS/ME).

When asked about symptom management, patients described how they had been left to find their own information and persuade the GP to meet their needs. This could be a confusing process as the internet could present contradictory, and sometimes unhelpful, messages on the treatment of CFS/ME:

*“so she knew she had ME… and then had to go and do your research and decide for yourself which treatments (were suitable)… it's just a shame that there isn't more (help) within…you know, within the system.”* Carer 9 (male, wife with moderate CFS/ME).

Patients and carers explained how they then took this information to their GP in an attempt to raise their awareness of the condition:

*“(The GP) did admit that he didn't know much about it because when I told him about the chronic fatigue service and various other things, he asked me for the information and he said would I bring it in so that he could pass it on to…for other patients as well. So he was quite open to knowing more about it really.”* Patient 12 (female, mild/moderate CFS/ME).

Some patients also sought help from private professionals or paid for alternative therapies as they did not feel that they received any support from the NHS. This often led to financial costs:

*“we started spending money blindly and I would do anything to get him better, so it’s like my priority was not…we don’t have holidays basically but want to get better so that life goes on. So we went to another private doctor. He used to do acupuncture,acupressure… Homeopathic. So it was like all that sort of thing. And we spent a lot of money there. Nothing is happening. Nothing is improving… By that time we spent about £20,000.”* Carer 5 (female, husband with severe CFS/ME).

This gap in knowledge was also recognised by CFS/ME specialists who highlighted a training need in primary care:

*“GPs and practice nurses want to do the best for their patients but often they don’t know what to do… we bandy around things like (sleep) hygiene and pacing and lifestyle management but do we actually know what we’re talking about, exactly what the narrative is in the consultation”* CFS/ME specialist 2.

However, some GPs did describe attempts to engage in symptom management with their patients. This included providing advice on pacing and graded exercise, and referring patients to services for interventions such as cognitive behavioural therapy (CBT). Although the need for a specialist services is recognised, health professionals agree that GPs can successfully recommend ways to manage many symptoms associated with CFS/ME:

*“cognitive therapies work, antidepressants work for some people, pacing undoubtedly works, changing people’s ideas about what they can do work, attending to their social and psychological situation helps.”* GP 1.

Patients and carers also highlighted the need for sign posting from their GP. Information on local support groups, advice on benefits and referrals to the third sector. However, most GPs and practice nurses did not have details of relevant contacts:

*“I don't know of any local support groups or anything. And I think it would be important for people to get in touch with other people that have had similar experiences, because I think that would help… I think I need advice where we should be referring to.”* PN4.

Health professionals described difficulties with referral to secondary care due to fragmented services and a lack of collaboration and a number of GPs and practice nurses were unaware of specialist CFS/ME services. Others had referred their patients to the specialist service, but lacked an understanding of what these services can offer patients. There was concern around the long waiting time for patients to attend the specialist service and it was suggested that improved communication between primary care and the specialist service may enable the GP to manage the patient during this period:

*“it’s been difficult to access the right services and support groups because they aren’t obvious. I mean I’ve never had a talk on ME and I go to every educational thing going. It wasn’t covered in the general practice nurse course that I did.”* PN2.

#### Implications for resource development

There is a need for GPs and practice nurses to develop an understanding of symptom management and appropriate sign posting for CFS/ME. GPs and practice nurses wanted to be able to print information on symptom management from an on-line resource during the consultation:

*“I think if it’s a brief one page (leaflet), you’re more likely to actually read it and possibly remember it and refer back to it. I think especially with CFS, I guess the concentration and you know energy required to sort of look at information and digest it, it would need to be brief snippets rather than lengthy tomes.”* PN1.

GPs also suggested that a DVD would be useful for those patients who would struggle to read written resources because of fatigue and concentration and memory problems:

“*I think a DVD would be useful for patients very much because they can take away something to actually look at.”* GP 6.

The patients and carers stated that a brief leaflet outlining the symptoms and the evidence for management options would be useful at diagnosis. They then suggested that the resources should give them the opportunity to go back to their GP to discuss each problem in more detail. For patients who might find it too difficult to read leaflets, a DVD, which could be watched with their family or carer would be preferred. As well as explaining symptom management, patients said that they would like an overview of the evidence base on the DVD and case studies of patients and carers to gain an understanding of how others live with the condition:

“*If you could get like a booklet or something (which includes) support, what things work, what things don’t work, and I think that would have been very, very, very helpful, definitely…”* Patient 14 (male, mild CFS/ME).

### Getting the most out of a consultation

Patients and carers described how visiting the GP can be a challenging experience. Patients described difficulty in remembering or articulating their symptoms and how they would take a carer or family member with them to help make the most of their consultation, and that a double appointment was sometimes needed. Some patients also highlighted a need for flexibility when making appointments:

“*(She) can suffer memory loss so I thought I always needed to be there just to prompt her in the background if there was anything she needed more to say… And emotionally, you know, she obviously felt quite emotional about it, particularly in the early days… Each visit is quite brief to the GP so (she) had to know in her own mind what she wants to say so… you've got to think of the right sort of questions really”* Carer 9 (male, wife with moderate CFS/ME).

Health professionals also recognised that a 10 minute consultation with a patient with CFS/ME can be a challenge due to the variety and complexity of symptoms:

*“your heart sinks when they come in the room really when you see… and they’ve got depression down as well because someone has given them anti-depressants and thought they were depressed with the symptoms before, and they’ve also got lots of other medical problems usually.”* GP 7.

A ten minute consultation was also seen as a potential barrier to diagnosis by CFS/ME specialists as GPs may not be able to gain a complete understanding of the variety of symptoms patients can experience, and impact of these symptoms on their life:

“*I think one of the problems is that GPs have such a short amount of time to see patients… people often just take their main problem, their one problem, and I think sometimes the whole picture can be missed if you’re not seeing somebody on a regular basis*.” CFS/ME specialist 2.

#### Implications for resource development

Patients need GPs and practice nurses to be aware of how the symptoms of CFS/ME can impact on their ability to articulate and remember information in a consultation. Patients may need extended appointments, flexibility, phone appointments, email correspondence with the GP, and an occasional home visit. In order to support the patient to get the most out of their consultation, guidance on how to prioritise their symptoms need to be provided in the patient resources.

### The role of carers

Patients and carers described the important role that carers play in the management of the illness. This included support in the home and during a GP consultation:

*“I do ask* [my mum] *for things and she'll come and she'll help me with the children, and she'll either take them out or have them stay at hers or something, so she helps me with the children a lot… I've already had complaints from my dad about my mum's always here and helping and things.”* Patient 1 (female, severe CFS/ME).

When taking on a caring role, carers explained how they could feel isolated as they had to devote so much time to the patient in order to attend to their needs. The financial pressure of the patient losing their job or paying for alternative therapies was also cited as a stressor:

*“And how are we going to manage? For god sake, we all need money. I mean am I going to get electricity free, gas free, what? And because* [my husband] *feels cold all the time, nearly £2,000 we spend on utilities, on gas and electricity, a year. That’s quite an expense because we have to keep the house warm.”* Carer 5 (female, husband with severe CFS/ME).

Despite the valuable role that carers can take in supporting a patient, some carers lacked understanding of CFS/ME and were frustrated by the patient’s symptoms, which in turn put pressure on the relationship. They also discussed the danger of giving inappropriate advice or support to patients, exacerbating symptoms by becoming over solicitous or pushing the patient to do too much too soon:

*“I had no idea what she was going through because you don’t understand you know you come home and she’s in bed.* [I used to say] *‘Come on get out of bed do something, you might feel better’… I had no idea. I pushed her, she got worse”* Carer 6 (male, wife with moderate/severe CFS/ME).

GPs and practice nurses stated that although their practice has a ‘carers register’, they did not recognise and register carers of people with CFS/ME:

*“It’s very difficult in a GP setting to chase the carers, it’s very difficult, and then if you chase them on their own confidentiality kicks in as well… There’s no formal assessment on carers’ needs in the truer sense, no, there’s no formal sense.”* GP 9.

#### Implications for resource development

GPs need to recognise the role of carers in supporting people with CFS/ME. The impact of this supporting role on the carer themselves should also be considered. Carers need information on CFS/ME and support groups to encourage a stable relationship between the carer and patient and to facilitate appropriate support.

A practitioner e-learning module should raise awareness of the needs of carers and provide practical advice on how to both support the carer and involve them in the management of the patient. A patient and carer DVD will provide carers with information on how to support both the patient and themselves.

## Discussion

### Summary of the main findings

This is the first study to systematically explore the perspectives of the range of stakeholders, using a ‘bottom-up’ approach and developing complementary resources to be used together to improve the management of people with CFS/ME, and improve primary care professionals’ confidence in managing people with this condition. Patients, carers and practitioners agreed that the diagnosis and management of CFS/ME are best placed in primary care, however, the importance of appropriate referral was also recognised. Patients and carers stressed the importance of recognising CFS/ME as a legitimate condition, and the need to be believed by health care professionals. A diagnosis of CFS/ME was one way of achieving this. In accordance with NICE guidelines [1], patients wanted a positive, early diagnosis and information on how to manage their symptoms. However, practitioners described difficulties in managing CFS/ME, and welcomed skill based training. An online approach was preferred as face to face training was thought to be too time consuming.

Patients and carers did not want to be over loaded with information at diagnosis, and asked for a leaflet with an overview of the symptoms of CFS/ME, the management options and useful links to be provided at diagnosis. They then suggested that further information on symptom management and relevant signposting should be given in short modules, over a number of consultations. Health professionals also requested information from a reliable source that could be printed from their computer. A set of A4 information leaflets were therefore developed, providing information on management options and useful websites and resources. These are based on existing materials, modified and extended in collaboration with the patient involvement groups. A DVD has also been made to enable information sharing with carers and family, and also for those whose symptoms act as a barrier to reading. These resources will be presented to the patient by the GP in a CFS/ME pack. Table 3 outlines how the resources developed have been designed to meet the needs identified by patients, carers and practitioners in this study.

**Table 3:** Patient, carer and practitioner resources

**The need identified**	**The resources developed to meet the need**
**The need to ensure that the patient feels believed**	• E-learning resource for GPs and other health care professionals, containing guidance and information on the evidence base (Appendix 1) as well as clips of consultations demonstrating skills that are helpful in managing patients with CFS/ME.
**The need for positive framing of the diagnosis**	• A brief information leaflet to be provided by the GP at diagnosis. This provides an overview of the symptoms of CFS/ME, the management options and useful websites and resources.
	• E-learning resource for GPs and other health care professionals, including guidance and information on how diagnose a patient with CFS/ME (Appendix 1).
	• Patient activity diary to be used by the GP to aid diagnosis.
**The need to define, prioritise and manage symptoms**	• A set of A4 information leaflets on the management of each of the main symptoms that can be printed from the practice computer (fatigue, sleep, pain, cognitive dysfunction/memory problems, managing setbacks, work and education, stress, anxiety and depression and diet and nutrition). As patients have stated that they can be overwhelmed by too much information this takes a modular approach. Patients will be encouraged to prioritise their symptoms and discuss management options at each consultation. The patient will be given a folder to then keep this information together.
	• DVD for patients and carers where the evidence base and symptom management modules will be presented by CFS/ME specialists. This was produced in order to overcome problems associated with CFS/ME in terms of concentration, and problems of language and poor health literacy (Appendix 2).
	• E-learning resource for GPs and other health care professionals, containing guidance and information on how to manage patients with CFS/ME (Appendix 1).
**The need to get the most out of the consultation**	• DVD for patients and carers with an example of how to prepare for a consultation and how to prioritise symptoms (Appendix 2).
	• E-learning resource for GPs and other health care professionals, containing guidance and information on how to support the patient and carer in the consultation (Appendix 1).
**The need to support carers**	• DVD for patients and carers which can be watched with the family. This includes information for carers and a real life account of a carers’ experience of caring for someone with CFS/ME (Appendix 2).
	• A4 information leaflet provides information for carers.
	• E-learning resource for GPs and other health care professionals, containing guidance and information on how to support carers (Appendix 1).

### Strengths and limitations of the study

A strength of this study is the patient and public involvement (PPI) that has been central to the development of the resources. By collaborating with patients and carers, we ensured that the research would reflect their needs and concerns. Patient and carer perspectives and priorities enhanced the study design, recruitment, data interpretation and development of the resources [30,31]. Recruitment of patient participants from CFS/ME support groups may have introduced bias in that these people may be particularly vocal about their condition and more dissatisfied with current care; however, by also recruiting using flyers and from third sector groups, we feel this potential bias is reduced.

By actively recruiting from BME groups, we aimed to ensure that the resources were culturally sensitive. However, it was difficult to recruit BME participants as many do not recognise CFS/ME as a medical condition [32]. The majority of those who were recruited were second or third generation, middle class immigrants who had English as their first language. Their resource needs reflected those of the White British population [33]. It is also important to note that the BME patients were South Asian, Black British or Indian so the views of other ethnic backgrounds are not represented. It is also important to note that patients had received their diagnosis between 6 months and 23 years prior to interview (mean = 8 years). As time goes on, people’s views on their CFS/ME and their assessment of their needs may change. Furthermore, interviews were only conducted in the North West and the chosen sites have a specialist CFS/ME service and active support groups.

We had difficulty recruiting Practice Nurses to be interviewed (n = 5) and reasons cited included workload, difficulty freeing the time up in their working day and level of reimbursement. This reflects the findings from previous work [18]: Practice Nurses may not see CFS/ME as a major part of their workload as they are focussed on chronic disease management for conditions that are incentivised by the Quality and Outcomes Framework (QOF) [15].

### Comparison with previous literature

The management of a patient with CFS/ME requires the health professional to employ a wide range of skills, such as engagement, problem solving and developing a model of the illness in collaboration with the patient [13]. Previous literature has highlighted a lack of GP confidence in the diagnosis and management of CFS/ME [6,13]. Bowen et al. [19] reports varying attitudes to CFS/ME as a legitimate condition which resonates with the findings of this study where patients stated that they need to be believed. Research also describes a lack of empathy, negative attitudes towards people with CFS/ME, and the belief that the label of CFS/ME could be potentially harmful for the patient [9,19]. We are not aware of any research that shows that making the diagnosis of CFS/ME leads to a worse outcome. Our previous work suggests that patients find the label of CFS/ME helpful, ending uncertainty, and the patients and carers interviewed in this study support this view. This supports the need for the educational resources and training developed in this study.

The needs of carers have been relatively understudied and little is known about the types of information and support that are valued by this group. This study highlighted the pressures of caring for a patient with CFS/ME and the impact of inadequate or inappropriate support. This is in line with research that has shown that carers tend to have low scores for both physical and mental health compared to the general population [34]. Reduced wellbeing and an inability to cope have been shown to compromise a carer's ability to care for the patient, which in turn might affect the patient's illness course [35,36]. Behaviours, described as ‘overly attentive’ or ‘neglectful’, by significant others are have been reported to be associated with patients’ reported pain behaviours and levels of depression, even when controlling for pain intensity and fatigue severity [35]. It is therefore important for GPs to offer information and support to carers, including the details of support organisations to help cope with emotional and financial pressures and advice on how to give appropriate support to patients.

## Conclusion

This paper describes the development of CFS/ME resources for use with patients with CFS/ME and their carers, and primary care practitioners. The process was truly collaborative, with input at all stages in the process from our patient co-investigator, the carer on the research management group and our two Patient Involvement Groups. In addition, data from qualitative interviews with patients, carers, primary care and specialist CFS/ME practitioners has contributed to the development of the resources. These resources aim to facilitate the self-management of symptoms, supporting patients to live the best possible quality of life and work towards recovery, and to assist GPs in feeling more confident in making the diagnosis of CFS/ME and supporting and managing patients with CFS/ME and their carers. The research team are now evaluating the usefulness, and acceptability of these resources from patient, carer and practitioner perspectives.

## Appendix A. Appendices 1 and 2 outline the content of the materials developed by the research team which are currently being evaluated for the acceptability

### A.1. Appendix 1

E-learning resource for GPs and other health care professionals, containing guidance and information on how to diagnose and manage patients with CFS/ME. This includes:

– Key messages from qualitative work talking to patients, carers and specialist practitioners, and distilling the available evidence.

– An overview of the evidence base.

– An overview of the symptoms of CFS/ME.

– A link to the NICE guidance.

– A description of how to make the diagnosis, and the importance of the diagnosis.

– Pod casts to show descriptions of what it’s like to have CFS/ME (with actors playing the role of a carer and patients with mild and moderate CFS/ME to preserve anonymity. The service user co-investigator in this study presented her experiences as a patient with severe CFS/ME).

– Sound bites of patients describing what it is like to experience the symptoms of CFS/ME.

– Information of the impact of CFS/ME, for patients, carers and the economy.

– Management options for: fatigue, sleep, pain, cognitive dysfunction/memory problems, stress and anxiety and diet and nutrition. This will provide advice on activity and rest, the idea of working towards recovery and includes guidance on referral and signposting to other services. Information can be printed off as A4 leaflets to give to patients.

– Supporting the patient to manage setbacks.

– Support for carers.

– Referral to specialist services.

– Health beliefs.

– Top tips from patients in managing their condition.

– Making the GP practice accessible to CFS/ME patients.

– The role of primary care in diagnosing and managing CFS/ME.

– Useful links and resources.

### A.2. Appendix 2

DVD for patients and carers includes:

– An overview of the evidence base.

– Accounts of what it is like to live with CFS/ME (with actors playing the role of a carer and patients with mild and moderate CFS/ME to preserve anonymity. The service user co-investigator in this study presented her experiences as a patient with severe CFS/ME).

– A list of symptoms with sound bites from patents describing what each symptom feels like for them.

– The consequences of CFS/ME.

– Information for carers.

– Information on what to expect from diagnosis and the activity diary.

– Management options for fatigue, sleep, pain, cognitive dysfunction/memory problems, stress and anxiety and diet and nutrition.

– Referral to the specialist CFS/ME team.

– Relaxation.

– Relapse prevention.

– Work and education.

– Role of the GP and practice nurse and how to prepare for a consultation.

– Top tips from other patients and carers and additional links and resources.

## Competing interests

The author(s) declare that they have no competing interests.

## Authors’ contributions

KH contributed to the recruitment of participants, patient and carer data collection, data analysis and drafted the paper. SP contributed to the design of the study, data analysis and writing the paper. LF contributed to the recruitment of participants, GP and practice nurse data collection, data analysis and writing the paper. LR designed and managed this qualitative study and contributed to the recruitment of participants, data analysis and writing the paper. AW contributed to the design of the study, data analysis and writing the paper. KL contributed to the design of the study, data analysis and writing the paper. PT contributed to the design of the study and contributed to the recruitment of participants and writing the paper. YL contributed to the recruitment of participants, checking the analysis and resources and writing the paper. CCG designed and managed this study. She contributed to the recruitment of participants, data collection, data analysis and writing the paper. CCG is guarantor for the study and paper. All authors read and approved the final manuscript.

## Authors’ information

Pam Turner is patient co-investigator and Yvonne Leech is carer representative.

## Pre-publication history

The pre-publication history for this paper can be accessed here:

http://www.biomedcentral.com/1471-2296/13/93/prepub
